# Subfamily-Specific Adaptations in the Structures of Two Penicillin-Binding Proteins from *Mycobacterium tuberculosis*

**DOI:** 10.1371/journal.pone.0116249

**Published:** 2014-12-31

**Authors:** Daniil M. Prigozhin, Inna V. Krieger, John P. Huizar, Daniela Mavrici, Geoffrey S. Waldo, Li-Wei Hung, James C. Sacchettini, Thomas C. Terwilliger, Tom Alber

**Affiliations:** 1 Department of Molecular and Cell Biology and California Institute for Quantitative Biosciences, University of California, Berkeley, California, 94720, United States of America; 2 Los Alamos National Laboratory, Los Alamos, New Mexico, 87545, United States of America; 3 Department of Biochemistry and Biophysics, Texas A & M University, College Station, Texas, 77843, United States of America; Institut Pasteur, France

## Abstract

Beta-lactam antibiotics target penicillin-binding proteins including several enzyme classes essential for bacterial cell-wall homeostasis. To better understand the functional and inhibitor-binding specificities of penicillin-binding proteins from the pathogen, *Mycobacterium tuberculosis*, we carried out structural and phylogenetic analysis of two predicted D,D-carboxypeptidases, Rv2911 and Rv3330. Optimization of Rv2911 for crystallization using directed evolution and the GFP folding reporter method yielded a soluble quadruple mutant. Structures of optimized Rv2911 bound to phenylmethylsulfonyl fluoride and Rv3330 bound to meropenem show that, in contrast to the nonspecific inhibitor, meropenem forms an extended interaction with the enzyme along a conserved surface. Phylogenetic analysis shows that Rv2911 and Rv3330 belong to different clades that emerged in Actinobacteria and are not represented in model organisms such as *Escherichia coli* and *Bacillus subtilis*. Clade-specific adaptations allow these enzymes to fulfill distinct physiological roles despite strict conservation of core catalytic residues. The characteristic differences include potential protein-protein interaction surfaces and specificity-determining residues surrounding the catalytic site. Overall, these structural insights lay the groundwork to develop improved beta-lactam therapeutics for tuberculosis.

## Introduction

*Mycobacterium tuberculosis* (*Mtb*), the etiologic agent of tuberculosis (TB), presents major threats to public health worldwide [1]. Emergence of *Mtb* strains resistant to the front-line and second-line drugs underscores the necessity of developing new therapeutics [2]. The mycobacterial cell wall, a complex structure responsible for mediating *Mtb* interactions with the environment, is a validated drug target [3]. The cell wall consists of several layers: peptidoglycan, arabinogalactan, mycolic acids, and the polysaccharide capsule [4]. Key anti-TB drugs target the biosynthesis of arabinogalactan and mycolic acids [3]. Beta-lactams, antibiotics that target peptidoglycan biosynthesis and are effective against diverse bacterial pathogens, have not yet been deployed against *Mtb* due to the presence of an efficient beta-lactamase. Nonetheless, a combination of meropenem, a beta-lactam, and clavulanate, a beta-lactamase inhibitor, is being tested as a treatment for both active and latent TB [5]. Genetic, biochemical, and structural characterization of enzymes involved in peptidoglycan homeostasis is critical for development of this and other new therapeutics.

Peptidoglycan, which forms the membrane-proximal layer of the mycobacterial cell wall, is composed of repeating disaccharide-pentapeptide units that form polysaccharide chains covalently crosslinked through the stem peptides [6]. In *Mtb*, the disaccharide is N-acetyl glucosamine N-glycolyl or N-acetyl muramic acid [7], and the pentapeptide attached to the lactate moiety of muramic acid is L-Ala-iso-D-Glu-meso-DAP-D-Ala-D-Ala (DAP stands for diaminopimelic acid) that is typically amidated on the free carboxyl groups of iso-D-Glu and DAP [8]. Beta-lactams inhibit several enzyme classes that catalyze different reactions on peptidoglycan substrates. These penicillin-binding proteins (PBPs) include D,D-transpeptidases, which crosslink the third and fourth amino acids in the pentapeptide stems (3–4 crosslinks) [9]. In addition to the synthetic enzymes, PBPs include hydrolytic D,D-carboxypeptidases and D,D-endopeptidases.

**Figure 1:**
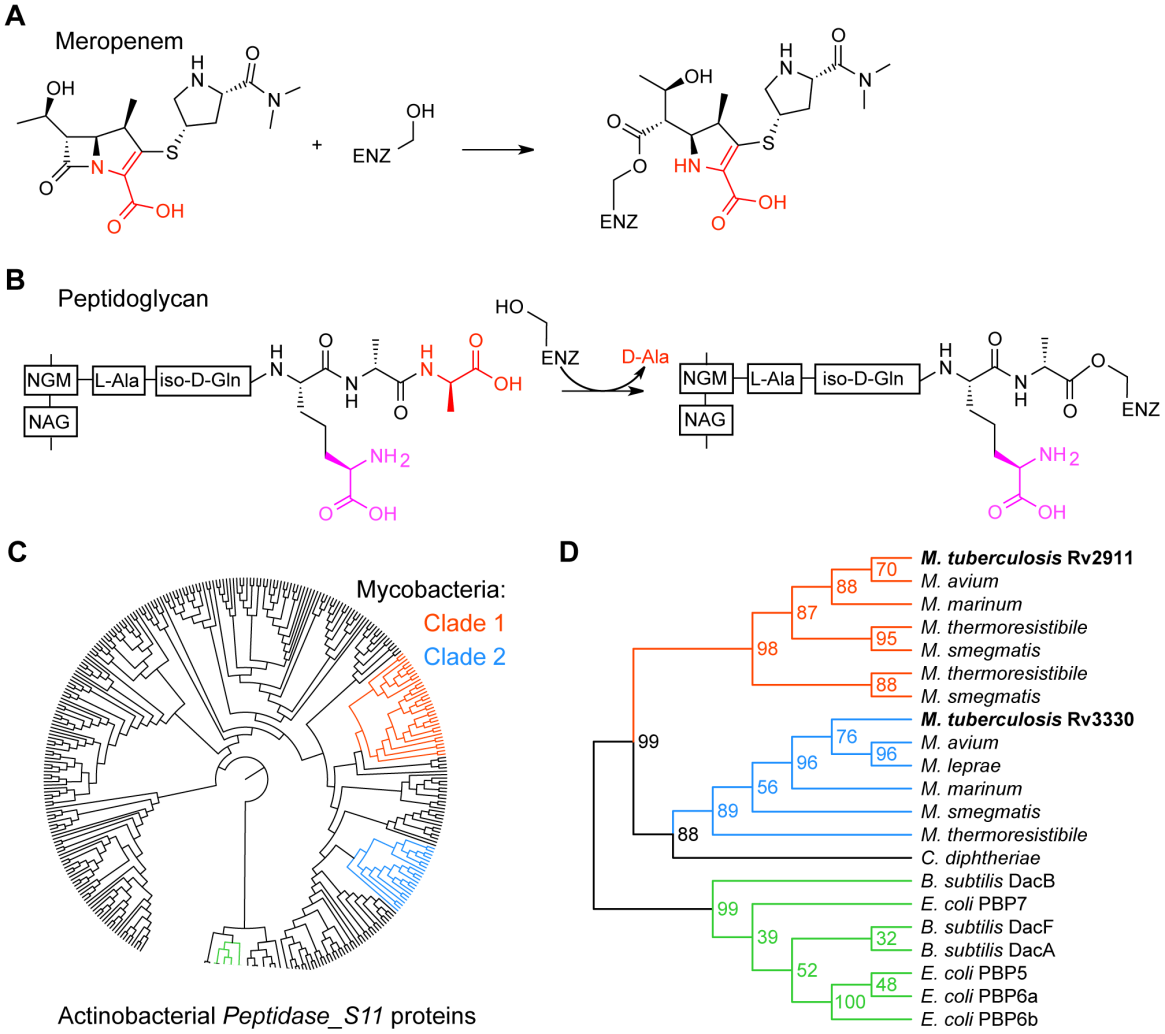
Activity and phylogeny of Rv2911 and Rv3330. A. The beta-lactam, meropenem, contains a D-Ala-like group (red) and irreversibly acylates penicillin-binding proteins, including Rv2911 and Rv3330. B. Enzyme acylation by peptidoglycan stem peptide, the first step in peptidoglycan carboxy- and trans-peptidation, highlighting the similarity of the terminal D-Ala leaving group (red) and the D-Ala-like acceptor moiety of DAP (purple). C. Neighbor-joining tree of *Peptidase_S11* family proteins of Actinobacteria fall into several clades. Of the two mycobacterial clades, Clade 1 (orange) contains Rv2911, and Clade 2 (blue) contains Rv3330. *E. coli* and *B. subtilis* Peptidase_S11 enzymes (green) form an out-group with several actinobacterial sequences. D. Maximum-likelihood tree of *Peptidase_S11* proteins from select mycobacterial species, *Corynebacterium diphtheriae*, *E. coli*, *and B. subtilis*. High bootstrapping values confirm the separation of mycobacterial sequences into two distinct clades.

Beta-lactams, including meropenem, mimic the terminal D-Ala-D-Ala dipeptide of the stem peptide and act as suicide inhibitors of penicillin-binding proteins (Fig. 1A). The reaction mechanisms of biosynthetic and hydrolytic penicillin-binding proteins share the first step. Attack of a serine nucleophile on the peptide bond between the two D-alanines of the donor stem leads to acylation of the enzyme by the penultimate D-Ala (position 4 in the stem peptide) and the release of the terminal D-Ala (Fig. 1B). Transpeptidases use a D-Ala-like distal end of the DAP residue (position 3 in the stem peptide) of the acceptor stem as a nucleophile to resolve the covalent intermediate, creating a 4-3 crosslink. In contrast, carboxypeptidases use a water molecule to resolve the acyl-enzyme intermediate, converting a pentapeptide stem to a tetrapeptide. Tetrapeptide formation not only regulates the degree of peptidoglycan crosslinking, but also provides substrate for the synthesis of 3-3 crosslinks essential for various physiological adaptations [10]. Compared to many beta-lactams, carbapenems appear to have a broader specificity. Meropenem, for example, is effective against penicillin-binding proteins as well as L,D-transpeptidases [11], an unrelated family of enzymes that form the 3-3 crosslinks. Investigation of changes in *Mtb* peptidoglycan structure in response to meropenem treatment suggested that the inhibitor not only blocks the transpeptidases that synthesize the peptidoglycan crosslinks, but also the carboxypeptidases, which trim the terminal D-Ala to form tetrapeptides [12].

Of the 11 predicted *Mtb* penicillin-binding proteins [9], only the synthetic enzyme PbpA (Rv0016c) has been structurally characterized [13]. The two penicillin-binding proteins in the *Peptidase_S11* Pfam family, Rv2911 (DacB2, UniProtKB Q7D6F2) and Rv3330 (DacB1, UniProtKB O53380), have recently been investigated for their potential role as major D,D-carboxypeptidases of *Mtb* [12]. Rv2911 is detected in *Mtb* culture filtrates [14]. Deletion and overexpression of *rv2911* are tolerated in culture [15]. Rv3330 and Rv2911 both demonstrate specificity for DAP-containing stem peptides [12].

To define the features that mediate inhibitor binding, we determined the crystal structures of a quadruple-mutant of Rv2911 in complex with a non-specific inhibitor phenylmethylsulfonyl fluoride (PMSF) and Rv3330 in complex with meropenem. The Rv3330-meropenem complex highlights an extended interaction surface that is distinct from that engaged by a donor peptide. Neither enzyme shows a strong binding affinity for peptidoglycan. In addition, the Rv2911 and Rv3330 structures reveal subfamily-specific conserved surfaces that distinguish these enzymes and are consistent with distinct functions in peptidoglycan homeostasis.

## Results

A search of the *Mtb* genome for *Peptidase_S11* Pfam family proteins (PF00768) yielded two hits: Rv2911 and Rv3330. Similar searches identified penicillin-binding proteins 5, 6a, 6b, and 7 in the model Gram-negative bacterium, *E. coli*, and DacA, DacB, and DacF in the model Gram-positive bacterium, *B. subtilis*. The neighbor-joining tree of unique actinobacterial *Peptidase_S11* sequences from the Pfam database showed at least five separate clades, one of which contained the *E. coli* and *B. subtilis* proteins included in the analysis. All mycobacterial sequences split into two clades, Clade 1 containing Rv2911 and Clade 2 containing Rv3330 peptidase (Fig. 1C). Bootstrapping analysis of the maximum likelihood tree of select mycobacterial species showed high confidence values for the two clades (Fig. 1D). Orthologs of both proteins were found in most sequenced mycobacteria. One exception was *M. leprae*, where the *rv2911* gene is inactivated. *M. smegmatis* and several other mycobacteria not only encode an *rv3330* ortholog, but also contain duplications of the *rv2911* gene. Based on this analysis, we concluded that the duplication event that allowed for diversification and specialization of Rv2911 and Rv3330 likely occurred in a common ancestor of all mycobacteria.

**Figure 2:**
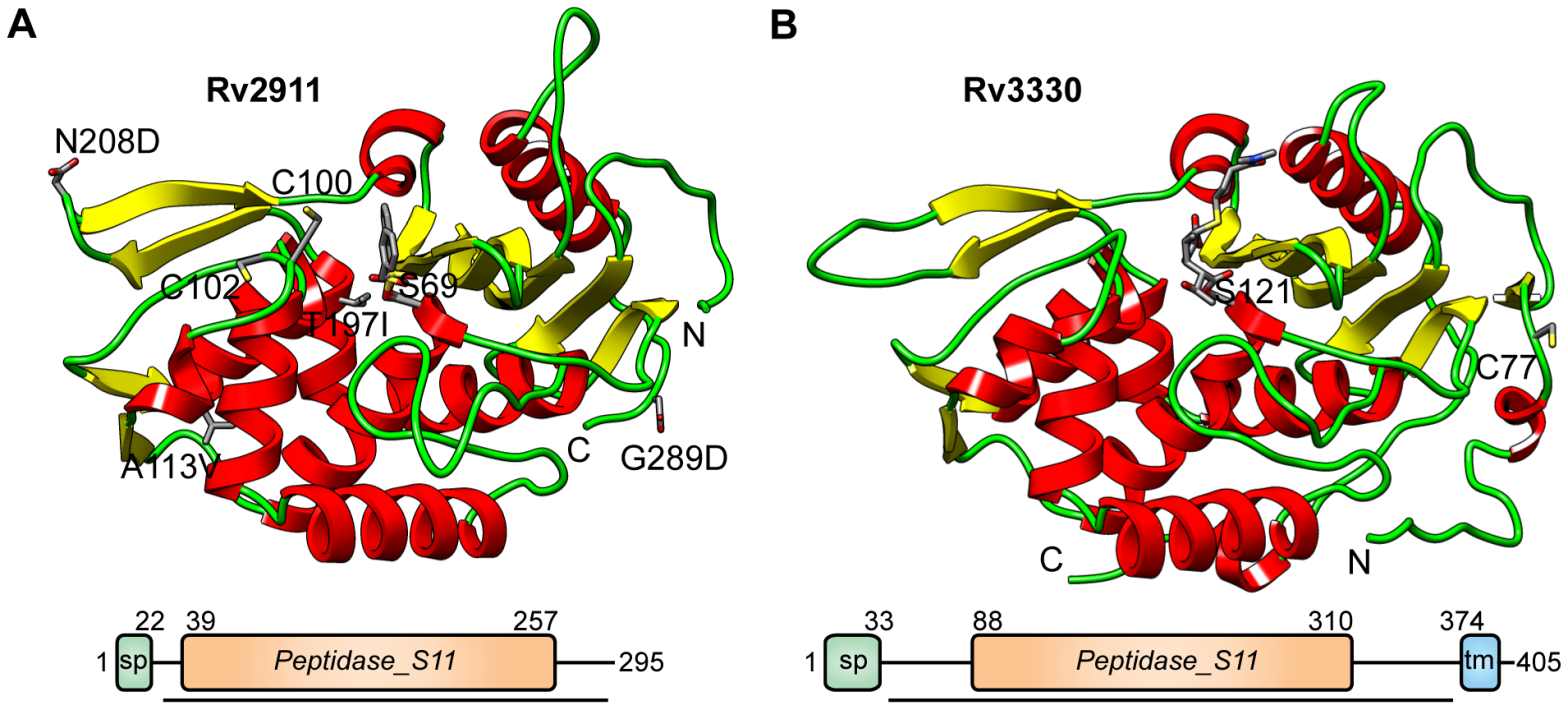
Structures and domain diagrams of Rv2911opt and Rv3330 enzymes. A. Rv2911_opt_ ribbon diagram showing the inhibitor, PMSF, bound to the catalytic Ser69 (sticks). Four mutations obtained during split-GFP optimization are highlighted along with two cysteines that participate in intermolecular disulfide bonds in the crystal. B. Ribbon diagram of Rv3330 structure showing meropenem bound to the catalytic Ser121 (sticks). Cys77, which forms an intermolecular disulfide bond in the crystals occurs in an N-terminal extension absent from Rv2911. In the domain diagrams, SP denotes the signal peptide, TM represents the trans-membrane helix, and numbers indicate sequence positions of domain borders. Sequences present in the crystallization constructs are underlined.

To investigate the biochemical and structural features of these proteins, we cloned, expressed in *E. coli*, and purified Rv2911 and Rv3330 peptidoglycan peptidases. Sequence analysis indicated that the two proteins share a similar architecture, containing predicted signal peptides at their N-termini and lacking the characteristic C-terminal domain of *E. coli* PBP5 (Fig. 2). A unique feature of Rv3330 is the presence of a single transmembrane helix at the C-terminus. The Rv3330 peptidase is thus predicted to be membrane associated, while Rv2911 is a periplasmic protein, consistent with its documented presence in *Mtb* culture filtrates [14].

Because the wild-type Rv2911 was poorly soluble and failed to crystallize, we undertook extensive construct optimization using the GFP folding reporter method [16]. Several rounds of directed evolution to improve solubility yielded an optimized gene sequence carrying six point mutations. Two of these mutations were in the predicted signal peptide region, which was truncated in further optimization. The resulting A113V, T197I, N208D, G289D quadruple-mutant protein was named Rv2911_opt_.

The Rv2911_opt_-PMSF structure was determined at 2.0 Å resolution using multiwavelength anomalous dispersion of Se-labeled enzyme. This structure was used as the search model to determine the Rv3330-meropenem structure, also at 2.0 Å resolution (Table 1). The structures showed similar overall folds containing two subdomains (Fig. 2). In the first subdomain, the β-sheet core is formed by strands from both N- and C-terminal sequences. The second subdomain is α-helical and contains two peripheral two-stranded β-sheets. Structural alignment of the two peptidases revealed a root-mean-square deviation (RMSD) of 0.76 Å over 235 Cα atoms, despite just 46% residue identity over the aligning region. The extended N-terminal sequences of Rv3330 add an extra strand to the β-sheet and a short peripheral helix together with a long coil. The Rv3330 C-terminal extension contains another short helix and extends away from the protein to make a crystal contact. Remarkably, crystals of both proteins were stabilized by disulfides across two-fold axes. Cys100 of Rv2911_opt_ was found in a disulfide with Cys102 from a symmetry-related molecule. In the Rv3330 crystals, Cys77 residues from adjacent molecules formed a disulfide across a two-fold axis. Of the four mutations in Rv2911_opt_, two, A113V and T197I, were buried, while the other two, N208D and G289D, were exposed and participated in a crystal contacts.

**Table 1:** Data collection and refinement statistics.

	Rv2911-PMSF	Rv3330-Meropenem
**Data Collection**		
Wavelength (Å)	0.980	1.116
Temperature (K)	100	100
Space group	H 32	P 41 2 2
Cell parameters		
*a* *b* *c* (Å)	75.13 75.13 230.44	76.291 76.291 99.978
α β γ (°)	90 90 120	90 90 90
Copies per a.s.u.	1	1
Resolution (Å)^a^	43.13-2.00 (2.07-2.00)	50.0 – 2.00 (2.07-2.00)
R_sym_ (%)	16 (56.7)	9.5 (80.4)
I/σI	7.7 (3.8)	19.9 (3.4)
Completeness (%)	99.8 (100)	100 (100)
Redundancy	14.9 (11.2)	14.0 (13.9)
**Refinement**		
Resolution (Å)	43.13 – 2.00	47.48-2.00
Number of reflections	32,821	20,595
R_work_/R_free_ (%)	17.3/19.9	18.35/21.04
Number of atoms		
Protein	1929	2214
Ligand	16	26
Solvent	150	144
B factors		
Protein (Å^2^)	23.14	33.10
Ligand (Å^2^)	22.7	43.10
Solvent (Å^2^)	29.74	37.70
RMSD		
Bond lengths (Å)	0.007	0.012
Bond angles (°)	1.1	0.99
Ramachandran plot		
Favored (%)	97	100
Outliers (%)	0	0
**PDB ID**	**4P0M**	**4PPR**

a Values in parentheses refer to the high-resolution shell.

**Figure 3:**
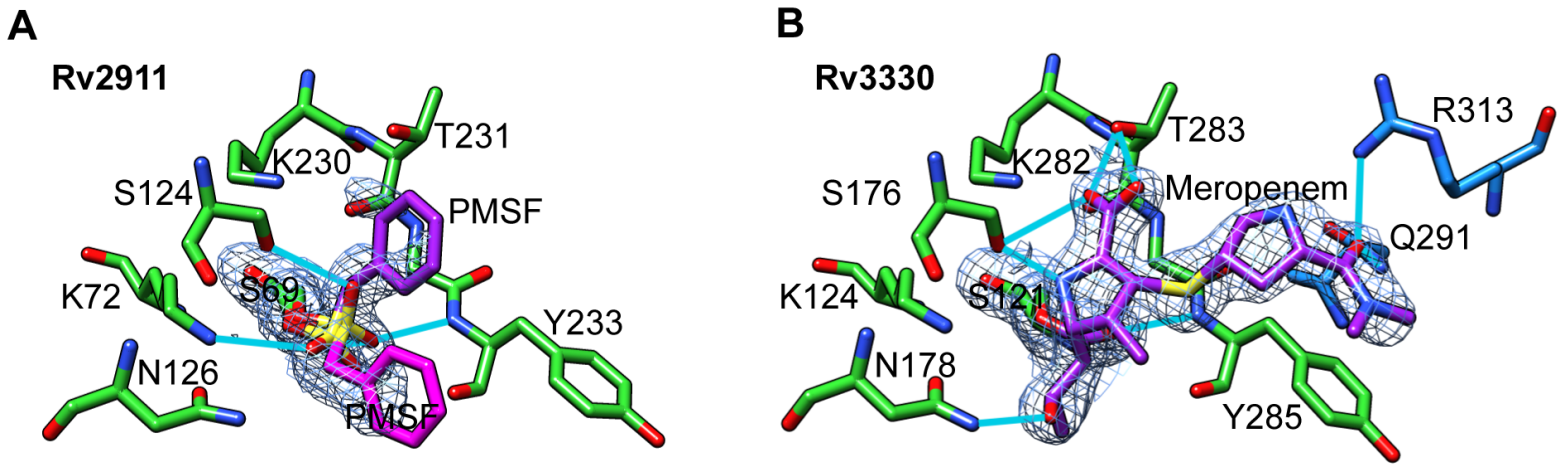
Conserved arrangement of catalytic-site residues of Rv2911 and Rv3330 peptidases. A. Rv2911opt-PMSF. Electron-density (2Fo-Fc, 2.0-Å resolution, 1σ) shows the inhibitor adopts two major conformations (purple and magenta). B. Rv3330-meropenem. Electron-density (2Fo-Fc, 2.0-Å resolution, 1σ) supports a unique model with extended interactions between the inhibitor (purple) and the enzyme. Common catalytic residues are colored green and Clade 2 conserved residues in Rv3330 are colored blue.

Both enzymes crystallized with covalently bound active-site inhibitors. Rv2911_opt_ was bound to a molecule of the nonspecific inhibitor, PMSF, carried over from the purification. Rv3330 was bound to meropenem, a beta-lactam antibiotic that was added prior to crystallization (Fig. 3). The active sites of both proteins contain the three typical penicillin-binding protein motifs: SXXK, SXN, and KTG. In both structures, the inhibitors were covalently linked to the serine of the first motif (STIK in Rv2911, SVIK in Rv3330). Residues of the other two motifs (SGN and KTGY in both proteins) interacted with the bound ligands. A common feature of both enzyme-inhibitor complexes is the interaction of either the carboxyl oxygen of meropenem or one of the sulfonyl group oxygens of PMSF with the oxyanion hole formed by the main-chain amides of the catalytic serine and the tyrosine of the third motif.

In the Rv2911_opt_-PMSF structure the conformation of the inhibitor was not unique, and the electron density for the phenyl group of the ligand suggested at least two alternate conformations. Neither conformation appeared stabilized by additional enzyme-inhibitor interactions (Fig. 3A). In contrast, upon binding to Rv3330, the meropenem molecule assumed a single conformation, as demonstrated by a well-defined electron density envelope extending away from the active site (Fig. 3B). This unique conformation is stabilized by several key interactions. First, the alanine-like moiety within meropenem is hydrogen bonded to Thr283 through the carboxyl and to Ser176 through both the carboxyl and amide groups. This interaction mimics the contacts made by the D-alanine leaving group of the natural pentapeptide substrate. Second, the sulfur of the thioester group packs against the main-chain atoms of Tyr284, with distances of approximately 3.7 Å to the nitrogen, oxygen, and the Cß atoms. Third, the π-system of the distal carbamoyl moiety participates in a stacking interaction with the phenyl ring of Tyr284. Finally, the oxygen of the carbamoyl group is hydrogen bonded to Asn291 and Arg313, two residues outside of the canonical active site motifs that are nonetheless universally conserved among mycobacterial orthologs of Rv3330.

**Figure 4:**
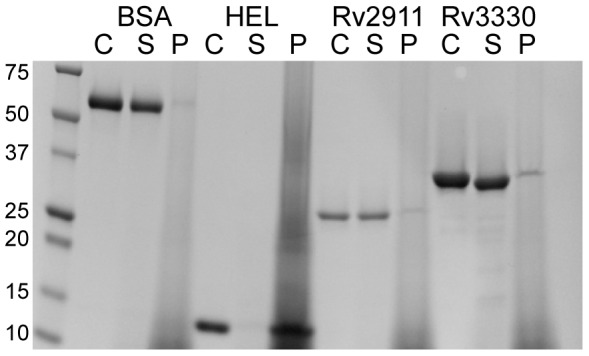
Rv2911 and Rv3330 catalytic domains bind weakly to peptidoglycan *in vitro*. SDS-PAGE of control (C), supernatant (S), and peptidoglycan fractions (P) following pull-down with *B. subtilis* peptidoglycan. Hen egg lysozyme (HEL) and bovine serum albumin (BSA) were used as positive and negative controls, respectively. The left lane contains molecular marker in kDa units.

To determine whether the catalytic domains bind polymerized peptidoglycan, we performed pull-down experiments with peptidoglycan from *B. subtilis*, which has a chemotype similar to peptidoglycan from *Mtb*. In contrast to hen egg-white lysozyme, a positive control that was retained in the peptidoglycan fraction, both Rv2911 and Rv3330 catalytic domains were found primarily in the soluble fraction (Fig. 4). This weak binding to peptidoglycan may reflect a requirement for additional protein-protein or protein-membrane interactions to properly localize and orient these peptidases relative to the substrate.

**Figure 5:**
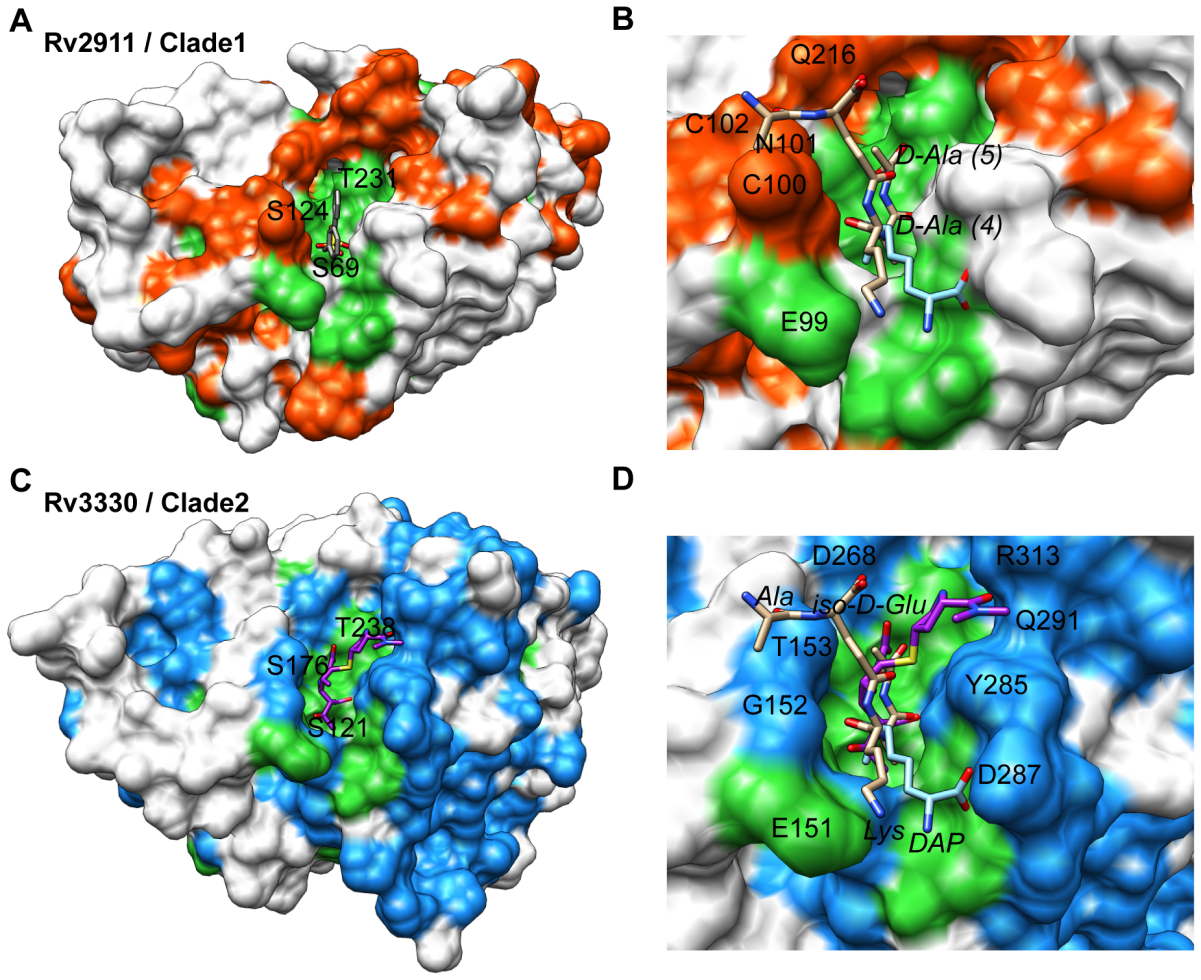
Different conserved surfaces surround the catalytic site in Clade 1 and Clade 2 D,D-carboxypeptidases. A. Surface representation of Rv2911_opt_-PMSF showing regions conserved across the two clades (green, 100% identity) as well as clade-specific conserved surfaces (orange, at least 90% identity within Clade 1). Surfaces corresponding to residues in the three conserved active site motifs are labeled to establish orientation. B. Catalytic site of Rv2911 (colored as in A) showing the superposition of stem-peptide mimicking ligands from *E. coli* PBP6 (tan, PDB 3ITB) and *B. subtilis* PBP4a (light blue, PDB 2J9P). The site that distinguishes lysine (tan) from DAP (light blue) is conserved at the bottom of the figure. PMSF was omitted for clarity. C. Rv3330-meropenem surface representation showing surfaces conserved across the two clades (green) and across Clade 2 (blue, at least 90% identity within Clade 2). A large surface (300 Å^2^) on the lower right in the figure is conserved in Clade 2 enzymes. D. The catalytic site of Rv3330 bound to meropenem (purple) with stem-peptide mimics from the superimposed complexes of *E. coli* PBP6 (tan, PDB 3ITB) and *B. subtilis* PBP4a (light blue, PDB 2J9P). The donor-peptide mimic (tan) exits the active site to the upper left of the figure. In contrast, meropenem interacts with a surface conserved in Clade 2 (upper right).

To determine the subset of residues that underpin penicillin-binding protein specialization, we constructed multiple sequence alignments of Clade 1 and Clade 2 proteins and mapped the conservation scores onto the protein surfaces (Fig. 5, A and C). To aid the interpretation of the observed differences, we overlaid peptidoglycan-mimicking ligands from *E. coli* PBP6 (PDB 3ITB) and *B. subtilis* PBP4a (DacC, PDB 2J9P) structures onto the *Mtb* peptidase surfaces (Fig. 5, B and D). Residues predicted to distinguish the stem-peptide meso-DAP, including Thr234, Leu166, and Asp99 (Rv2911 sequence numbering), are conserved across the two clades along with the catalytic center residues.

In contrast, clade-specific surface residues surround the catalytic centers of both enzymes. In Rv2911, these characteristic residues include the 100-CysAsnCys-102 triad on one edge of the catalytic site, as well as Gln216 on an adjacent side. The equivalent motifs in Rv3330 are 152-GlyThr-153 and Asp268, respectively. In addition, the extended meropenem-binding site of Rv3330 is distinct from that occupied by the donor peptide (Fig. 5D). Away from the active site of Rv3330, a surface region that centers on the loop containing 116-ArgHisArg-118 is conserved only in Clade 2 (Fig. 5C). This conserved surface measures approximately 300 Å^2^ and participates in a crystal contact that includes the intermolecular disulfide bond. The conservation of this area could indicate an interaction site with other proteins or with cell-wall constituents.

## Discussion

Investigation of mycobacterial peptidoglycan biology can potentially lead to improvements to current anti-TB therapies. The recent suggestion that a combination of meropenem with clavulanate can be effective at treating drug-resistant TB [5] has spurred interest in *Mtb* penicillin-binding proteins and L,D-transpeptidases. The latter enzymes were previously thought to be insensitive to beta-lactams, but Cordillot et al. showed robust inhibition of four out of five *Mtb* L,D-transpeptidases by four carbapenems, including meropenem [11]. The realization that broad target specificity can mediate drug potency [17] intensifies the need to understand carbapenem recognition by different target families. Here, we presented structural and phylogenetic analyses of two meropenem-sensitive *Mtb* penicillin-binding proteins, Rv2911 and Rv3330.

Poor solubility often constitutes a major challenge to structural and biochemical characterization of cell wall-associated enzymes. In this study, structure determination of wild-type Rv2911 protein could not be achieved. To overcome this challenge, we utilized an unbiased directed-evolution approach. Four point mutations discovered using directed evolution and the GFP folding reporter method resulted in the Rv2911_opt_ protein, which showed improved solubility and allowed crystallographic structure determination. Application of directed evolution methods to cell-wall associated proteins can aid research progress in this important area.

Structural analysis can be used to interpret biochemical observations such as Rv2911 and Rv3330 specificity for DAP-containing stem peptides. Based on superposition with structures of *E. coli* [18] and *B. subtilis* [19] penicillin-binding proteins bound to stem-peptide mimics we infer that the conserved SGN motif of the *Mtb* enzymes forms hydrogen bonds to the proximal carboxyl of DAP. Binding of the aliphatic middle part of the DAP residue occurs in a narrow groove that extends away from the catalytic serine between the side chains of conserved Thr234/286 (Rv2911/Rv3330) and Leu166/218. It is likely that a conserved Glu99/151 binds to the DAP amide group. The main-chain amides of residues in the loop that follows the KTGY β-strand are positioned to interact with the distal DAP carboxyl. In contrast to these shared features of the DAP-binding site, the L-Ala and iso-D-Gln residues of the superimposed stem peptide are positioned to interact with clade-specific 100-CysAsnCys-102 in Rv2911 and 152-GlyThr-153 in Rv3330.

The structure of the meropenem adduct of Rv3330 provides insights into the interactions of this inhibitor with the target enzyme. The beta-lactam core of meropenem interacts with the three characteristic penicillin-binding protein active-site sequence motifs. In contrast, the distal end of this inhibitor, which is variable in carbapenems, points away from the stem peptide-binding site (Fig. 5). The meropenem distal end binds a surface that is partially varied between Rv2911 and Rv3330. This binding mode explains the meropenem inhibition of these enzymes and suggests that the distal interactions of the drug could be exploited to mediate selectivity.

The phylogenetic analysis of the *Peptidase_S11* family proteins shows not only that Actinobacteria contain a set of penicillin-binding proteins different from that of Proteobacteria or Firmicutes, but also that gene duplication and specialization in mycobacteria produced two clades represented by Rv2911 and Rv3330. These proteins lack the characteristic PBP5 C-terminal domain that is widespread in both Proteobacteria and Firmicutes, supporting their classification as class 7 penicillin-binding proteins. This analysis contradicts the view that Rv3330 is a class 5 penicillin-binding protein [9]. The clade-specific residues in Rv2911 and Rv3330 define sequences that can mediate functional specializations.

The Rv2911_opt_ and Rv3330 inhibitor-complex structures provide a basis to interpret adaptations that occurred in the two peptidase subfamilies. Importantly, unlike comparisons of penicillin-binding proteins from different species, the Rv2911 and Rv3330 comparison excludes the possible effects of differences in peptidoglycan chemotype or other cell-wall features. Key subfamily adaptations include clade-specific residues surrounding the catalytic center. In addition, Rv3330 contains an extended conserved surface located away from the active site, and a conserved trans-membrane helix. These features suggest that the two peptidases could have distinct catalytic activities, different protein partners, or different modes of interaction with cell-wall polymers. In model organisms, penicillin-binding proteins interact with other peptidoglycan hydrolases and with dedicated regulatory proteins [20]. These interactions are critical to the functions of the cell wall remodeling complexes, including divisome and elongasome complexes that are responsible, respectively, for daughter cell separation and cell growth [21]. Discovery of peptidoglycan-processing complexes of *Mtb* as well as detailed biochemical analysis of substrate specificity should help reveal the overall logic of *Mtb* cell-wall homeostasis and point out the vulnerabilities that can be attacked by novel and updated drugs.

## Materials and Methods

### Bioinformatic analyses

Sequences of *Peptidase_S11* (PF00768) family proteins from Actinobacteria (taxid:1760) and Mycobacteria (taxid:1763) were downloaded from the Pfam server [22]. Sequences of homologous proteins from *E. coli* and *B. subtilis* were obtained from the UniProt database. Alignment of protein sequences truncated to the *Peptidase_S11* catalytic domain were generated with the *hmmalign* program, part of the HMMER package [23]. Belvu multiple-alignment editor was used to discard columns in the alignment that contained greater than 50% gap characters. Thereafter, redundant rows and rows containing more than 30% gaps were also removed. The edited alignment was analyzed using the PHYLIP package. The neighbor-joining tree of all actinobacterial and the maximum-likelihood tree of the selected mycobacterial proteins were generated using default parameters with 100 bootstrap replicates. The trees were analyzed and rendered using the FigTree program.

Clade 1 and Clade 2 proteins were gathered using BLAST search against mycobacterial genomes (taxid:1763) with either Rv2911 (Clade 1) or Rv3330 (Clade 2) as query. The BLAST results were first filtered based on percent query coverage (95% minimum) and then based on presence of Clade 2 characteristic transmembrane domains using TMHMM [24]. N-terminal signal peptide sequences were removed to generate predicted mature forms using the Signal-P web server [25], keeping the trans-membrane sequences intact. Alignments of clade-assigned sequences were generated using the MUSCLE program [26].

### Construct design, cloning, and protein purification

Maximal constructs matching the predicted mature forms were used: 26–291 for Rv2911 and 38–368 for Rv3330. This Rv3330 construct stopped at the beginning of the predicted C-terminal trans-membrane domain. The two genes were cloned from *Mtb* strain H37Rv genomic DNA into pDEST15 vector using the Gateway system (Invitrogen). Rosetta 2(DE3) *E. coli* expression cells (Novagen) transformed with GST-tagged TEV-cleavable constructs were inoculated into Terrific Broth, grown to OD_600_ of 0.6, and induced with 0.2 mM IPTG. Cells were grown with shaking at room temperature for 18 hours and harvested by centrifugation. Cell pellets were resuspended in 300 mM NaCl, 20 mM HEPES, pH 7.5, 0.5 mM TCEP, 10% glycerol and lysed by sonication at 4°C. Lysates were cleared by centrifugation and filtration using 0.22 µm filters. Samples were applied to equilibrated glutathione-affinity columns (GE), washed and then eluted with 20 mM reduced glutathione (Sigma) in the above buffer. Overnight dialysis into the glutathione-free buffer was performed following addition of 0.5 mg of TEV protease. Glutathione-affinity chromatography was repeated to remove the GST tag. Size-exclusion chromatography on a Superdex-75 column (GE) was used to complete purification. The proteins were used in peptidoglycan-binding assays and to obtain crystals of Rv3330. The Rv2911 construct used for crystallization, however, required extensive optimization as described below.

### Rv2911 cloning and construct optimization by directed evolution and the GFP folding reporter method

Full-length wild-type Rv2911, including a predicted leader sequence, was expressed from pET28 vector in *E. coli* BL21(DE3) cells at either 27°C or 37°C and was insoluble. The wild-type Rv2911 was subjected to forward evolution using the GFP folding reporter method [16]. During each round of evolution, 40 brightest colonies out of approximately 40,000 were picked for subsequent recombination by gene shuffling [16], [27]. After four rounds of forward evolution, there was no further increase in GFP fusion fluorescence. Following two rounds of backcrossing, the optimized sequence contained C12R, V18D, A113V, T197I, N208D, and G289D mutations. Because the soluble full-length protein failed to yield diffraction-quality crystals, an N-terminal truncation lacking 16-residue leader sequence was used.

### Rv2911_opt_ protein purification

*E. coli* BL21(DE3) cells transformed with C-terminally His-tagged Rv2911_opt_ expression plasmid were grown to OD600 of 0.6 and induced with 1 mM IPTG. Growth was continued at 22°C for 20 hours. The cell pellets were resuspended in buffer A (20 mM Tris, pH 8.0, 100 mM NaCl, protease inhibitor cocktail) and lysed by sonication. After ultracentrifugation for 30 min at 38,000 rpm, the supernatant was loaded on a 5 mL Talon superflow affinity column (Clontech) equilibrated with buffer A. After washing with 30 mL buffer A, the His-tagged Rv2911_opt_ was eluted from the Talon affinity column using buffer B (20 mM Tris, pH 8.0, 100 mM NaCl, 300 mM imidazole). The eluted fraction was dialyzed against buffer A and purified by gel filtration on a Superdex-75 column (Amersham Pharmacia Biotech) using buffer A with 10 mM β-mercaptoethanol for equilibration and elution. Selenomethionyl Rv2911 was prepared using methionine pathway inhibition method [28] by growing cells in presence of 60 mg/L L-Se-Met with additional 100 mg/L of threonine, lysine and phenylalanine each, and 50 mg/L of isoleucine, leucine and valine each. Mass spectrometry (Applied Biosystems) was used to confirm, that all 7 Met residues were replaced by Se-Met.

### Structure of Rv2911_opt_

Rv2911_opt_ crystals were obtained by mixing protein (15 mg/ml) 1∶1 with the mother liquor (17% PEG monomethyl ester 5000, 0.15 M Ammonium sulfate, 0.1 M Citrate buffer pH 5.6) in hanging-drop format at 22°C. The three-wavelength multiwavelength anomalous diffraction data of Rv2911_opt_ SeMet crystal were collected at Beamline 5.0.2 of the Advanced Light Source (ALS) at Lawrence Berkeley National Laboratory (LBNL), and processed with the HKL2000 package [29]. The structure was determined with SHELX [30] and the model was partially traced by RESOLVE [31] after solvent flattering with DM [32] in CCP4 package [33]. Tracing was completed with Arp/wArp [34]. PMSF moiety covalently linked to Ser69 was traced manually using SEB (o-benzylsulfonyl-serine) residue from PDB entry 1ZIY as a starting model. The program Coot [35] was used for manual rebuilding. Refinement was carried out using the program Phenix [36]. The structure has been deposited to the Protein Data Bank under accession number 4P0M.

### Structure of Rv3330

Rv3330 (10 mg/ml) was incubated with a two-fold molar excess of meropenem (Sigma) for 30 min and mixed 1∶1 with 0.1 M NaCl, 0.1 M HEPES, pH 7.23, 1.7 M ammonium sulfate well solution in a hanging-drop format. The crystals were cryopreserved in 20% xylitol in well solution and frozen in liquid nitrogen. X-ray diffraction data were collected under cryogenic conditions at Beamline 8.3.1 at the ALS at LBNL [37] and processed using HKL2000 [29]. The structure was determined by molecular replacement using the Rv2911_opt_ coordinates as the search model. Molecular replacement, model building, refinement, and validation were performed using Phenix [36] and Coot [35]. The structure has been deposited to the Protein Data Bank under accession number 4PPR. Molecular images were generated using Chimera [38].

### Peptidoglycan binding assay

One milligram of *B. subtilis* peptidoglycan (Sigma) was washed twice in 1 ml of pure water and resuspended in 100 µl of binding buffer (150 mM NaCl, 25 mM HEPES, pH 7). Protein samples (10 µl at 0.4 mg/ml) were added to 10 µl of peptidoglycan suspension, mixed, and incubated for 5 min at room temperature with nutation. Samples were centrifuged for two minutes at 11,500 g. Input control, bound, and free protein fractions were analyzed by SDS-PAGE and stained with Coomassie Brilliant Blue (Bio-Rad).
